# Creating a mouse model resistant to induced ischemic stroke and cardiovascular damage

**DOI:** 10.1038/s41598-018-19661-y

**Published:** 2018-01-26

**Authors:** Qing-Lan Ling, Anita J. Mohite, Emma Murdoch, Hironari Akasaka, Qun-Ying Li, Shui-Ping So, Ke-He Ruan

**Affiliations:** 1 The Center for Experimental Therapeutics and Pharmacoinformatics, Department of Pharmacological and Pharmaceutical Sciences, College of Pharmacy, University of Houston, Houston, Texas 77204 USA; 2 Department of Ultrasound, Union Hospital, Tongji Medical College, Huazhong University of Science and Technology, Hubei Province Key Laboratory of Molecular Imaging, Wuhan, China

## Abstract

Vascular prostanoids, isomerized from an intermediate prostaglandin (PG), H_2_, produced by cyclooxygenase (COX), exert various effects on the vascular system, both protective and destructive. During endothelial dysfunction, vascular protector prostacyclin/prostaglandin I_2_ (PGI_2_) is decreased, while inflammatory PGE_2_ and thrombotic TXA_2_ are increased. Therefore, our research aim was to reverse the event by controlling PGH_2_ metabolism by generating an *in vivo* model via enzymatic engineering of COX-1 and prostacyclin synthase (PGIS). The COX-1 and PGIS genes were linked to a 10-residue amino acid linker to form a Single-chain Enzyme Complex (SCHEC), COX-1-10aa-PGIS. Transgenic (CP-Tg) mice in a FVB/N background were generated using the pronuclear microinjection method. We first confirmed mRNA and protein expression of COX-1-10aa-PGIS in various CP-Tg mouse tissues, as well as upregulation of circulating PGI_2_. We then examined the cardiovascular function of these mice. Our CP-Tg mice exhibited marked resistance to vascular assault through induced carotid arterial blockage, acute thrombotic stroke and arterial arrest, angiotensin-induced peripheral vasoconstriction, and hepatic lipid accumulation after receiving a high-fat diet. They also had a longer lifespan compared with wild-type mice. This study raises the possibility of fighting cardiovascular diseases by regulating cellular arachidonic acid-derived PGH_2_ metabolites using enzymatic engineering.

## Introduction

Cellular prostanoid biosynthesis is regulated by cyclooxygenase (COX) enzymes, which exist in two isoforms (COX-1 and COX-2), and their downstream synthases. COX enzymes convert arachidonic acid (AA) into the unstable molecule prostaglandin H_2_ (PGH_2_), which must then be converted into biologically active prostanoids by downstream individual prostanoid synthases^1,2^. The single polypeptide chains of COX enzymes include two active sites; the first contains a tyrosine residue, which, via the cyclooxygenase reaction, produces prostaglandin G_2_ (PGG_2_) from AA. The second contains a heme, which through a peroxidase reaction, reduces PGG_2_ to PGH_2_, which is further transferred to downstream synthases to be isomerized to the final and active prostanoids^2^. For example, thromboxane A_2_ (TXA_2_) is produced via TXA_2_ synthase (TXAS), prostacyclin (PGI_2_) is produced via PGI_2_ synthase (PGIS), and prostaglandin E_2_ is produced via three PGE_2_ synthases. TXA_2_ production results in pro-thrombotic effects and vasoconstriction, while PGI_2_ production results in vasodilation, anti-thrombotic, and other vascular protective effects. Excessive PGE_2_, particularly PGE_2_ produced by inducible microsomal PGE_2_ synthase-1, is one of the major causative factors of vascular inflammation^1,3^. During endothelial dysfunction (one of the key factors leading to vascular diseases), the reduction of PGI_2_ associated with excessive production of inflammatory PGE_2_ and thrombotic TXA_2_ are major pathophysiological mechanisms underlying vascular and heart diseases such as stroke, hypertension, atherosclerosis, coronary arterial heart disease, and pulmonary arterial hypertension^4–6^. Using gene-knockout approaches, decreased biosynthesis of PGI_2_ together with increasing TXA_2_ has been shown to increase the risk of stroke and heart attack^7–9^. In addition, recent increases in the number of heart attacks caused by the use of COX-2 inhibitors (such as Vioxx) have been attributed to their effects on reducing PGI_2_ and increasing TXA_2_ biosyntheses in the vascular wall and platelets, respectively^3^. Thus, PGI_2_ is considered one of the most important vascular protectors^10,11^. Therefore, increasing the conversion of AA to PGI_2_, associated with the prevention of its conversion to thrombotic TXA_2_ and inflammatory PGE_2_, could provide a fundamental approach to restore endothelial dysfunction for the prevention and treatment of cardiovascular diseases. To achieve this goal, we recently employed an enzyme clustering method, in which we channeled PGH_2_ by linking COX-1 enzyme and PGIS enzyme to a well-defined 10-residue amino acid linker^10^. With the enzyme complex, we were able to regulate prostanoid biosynthesis in favor of PGI_2_ rather than TXA_2_ and PGE_2_ in cells. The varied cells, including COS-7, HEK293^11,12^, primary cultured adipose stromal cells^13^ and endothelial progenitor cells^14^, were transfected with the single cDNA of COX-1-10aa-PGIS or COX-2-10aa-PGIS. They showed high expression levels of the active hybrid enzyme with triple catalytic activities (converting AA into PGG_2_, then PGH_2_ and finally into active PGI_2_), which resulted in redirecting the conversion of endogenous AA metabolites more toward the favorable PGI_2_ production rather than the unfavorable TXA_2_ and PGE_2_ production^11–13^. These *in vitro* molecular and cellular studies, and preliminary experiments^15^ have provided a basis for us to hypothesize that the single gene of the hybrid enzyme could reduce the risk of heart disease *in vivo*. In this study, we integrated the protein engineering, enzyme complex construction, and transgenic mouse technique to gain primary control of cellular AA metabolism in favor of PGI_2_ biosynthesis and to disfavor PGE_2_ and TXA_2_ biosynthesis *in vivo*. The study was proposed in a previous editorial paper^15^. Here, with comprehensive experiments and more data, we demonstrated that the transgenic mouse model exhibited marked resistance to vascular assault through induced carotid arterial blockage, induced acute thrombotic stroke and arterial arrest, and angiotensin-induced peripheral vasoconstriction. The safety of the engineered trans-gene, COX-1-10aa-PGIS (integrated COX-1 and PGIS activities together in a single polypeptide chain) was confirmed by healthy animal development and maintenance of a longer lifespan with improved mental performance, which has been demonstrated in a previous publication^16^. It greatly increased the possibility of correcting inherited heart diseases using emerging gene medicine, such as the transgene, COX-1-10aa-PGIS, at the current experimental level.

## Results

### Generation of transgenic mice carrying a SCHEC, COX-1-10aa-PGIS gene

**Figure 1:**
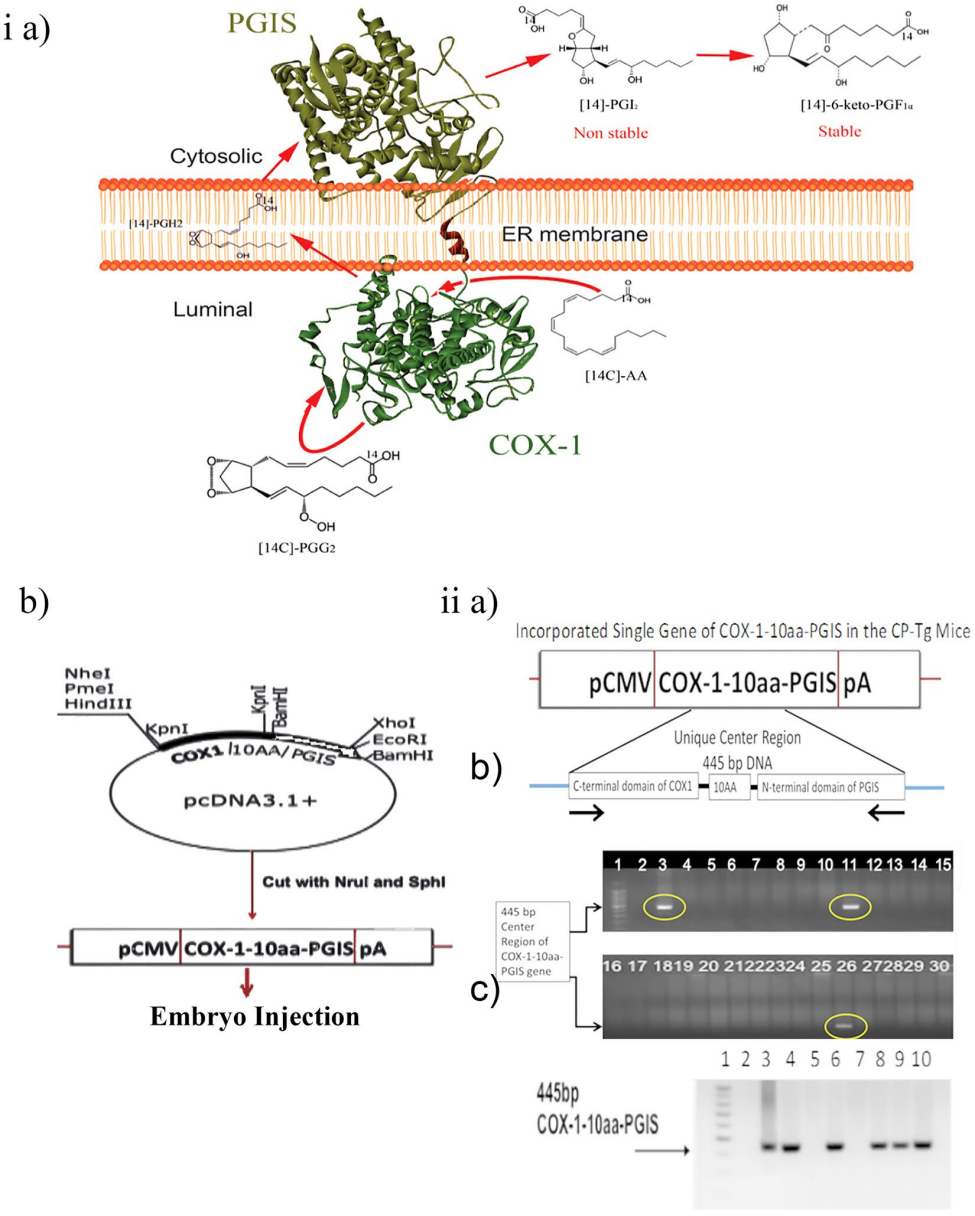
Mouse model generation and identification. (**ia**) Schematic presentation of the engineered SCHEC, COX-1-10aa-PGIS, with high efficiency in directly converting cellular AA to PGI_2_ on ER membrane by three continuous catalytic reactions within a single polypeptide chain. (**ib**) Construction of COX-1-10aa-PGIS cDNA. The sequence of COX-1 linked to PGIS through a 10-aa linker (COX-1-10aa-PGIS) was generated by PCR and subcloning procedures by the vector company (Invitrogen). The resulting cDNA was successfully subcloned into the pcDNA 3.1 vectors at Kpnl and BamHl sites containing a cytomegalovirus early promoter by PCR. The cDNA fragment (3.2 kb) containing COX-1-10aa-PGIS, a pCMV promoter and a PGH polyadenylation site (pA) was cut at the Nrul and Sphl sites from the COX-1-10aa-PGIS-pcDNA3.1 vector and purified to a single band as a transgenic gene for preparation of the transgenic mice. (**iia**) PCR primers designed to examine integration of the gene, a unique DNA fragment of 445 bp, into the genome. (**iib**) Genotyping of the first founders of the transgenic mice by PCR. The incorporated DNA of COX-1-10aa-PGIS in the mice was determined by PCR using the primers described in (**iia**), mouse tail tissues as template, and analysis by agarose gel electrophoresis to identify the specific 445-bp hybrid enzyme. (**iic**) PCR analysis of the second group of transgenic mice using the tail cut PCR approach in an identical manner to the procedures described in the (**iib**) panel.

We first demonstrated that the microenvironment of cellular prostanoid biosynthesis in primary cells could be controlled by our SCHEC, COX-1-10aa-PGIS (Fig. 1ia). Arachidonic acid metabolism was redirected in favor of PGI_2_ biosynthesis and disfavor to inflammatory PGE_2_ and thrombotic TXA_2_ biosynthesis for vascular protection, which was demonstrated *in vitro* and *in vivo* in our previous publication^15^. These data led to the hypothesis that the symptoms of cardiovascular diseases could be reduced by expression of a single gene, COX-1-10aa-PGIS, *in vivo*. To test this hypothesis, the COX-1-10aa-PGIS cDNA with a pCMV promoter for general expression and a pA segment were isolated from the COX-1-10aa-PGIS pcDNA3.1 vector (Fig. 1ib). Next, the founders of the original pair of COX-1-10aa-PGIS transgenic mice (CP-Tg mouse/mice) were created by pronuclear microinjection technology using the isolated COX-1-10aa-PGIS cDNA (Supplementary Fig. 1).

### Identification of the CP-Tg mice permanently incorporated with the single gene of COX-1-10aa-PGIS

A special set of polymerase chain reaction (PCR) primers was designed to determine the presence of the human COX-1-10aa-PGIS gene in the CP-Tg mouse chromosome. The primers contained the 3′- and 5′-ends of a unique DNA sequence of the center region of the incorporated single gene of COX-1-10aa-PGIS (from the C-terminal domain of COX-1 to the 10-aa linker and the N-terminal domain of PGIS) comprising a total of 445 bp (Fig. 1iia). Of the first 29 newborns, three founders (Fig. 1iib, lanes: 3, 11 and 26) permanently carrying the COX-1-10aa-PGIS gene were identified from mouse tail DNA by the tail cut-PCR approach using the designed primers. The founders were further bred with wild-type FVB/N mice and found to pass the transgene following Mendelian rules. The resulting seven heterozygous mice were further identified by the tail cut PCR approach (Fig. 1iic, lanes 3, 4, 6, 8, 9 and 10).

### The gene profile of COX-1-10aa-PGIS expression in different organs

**Figure 2:**
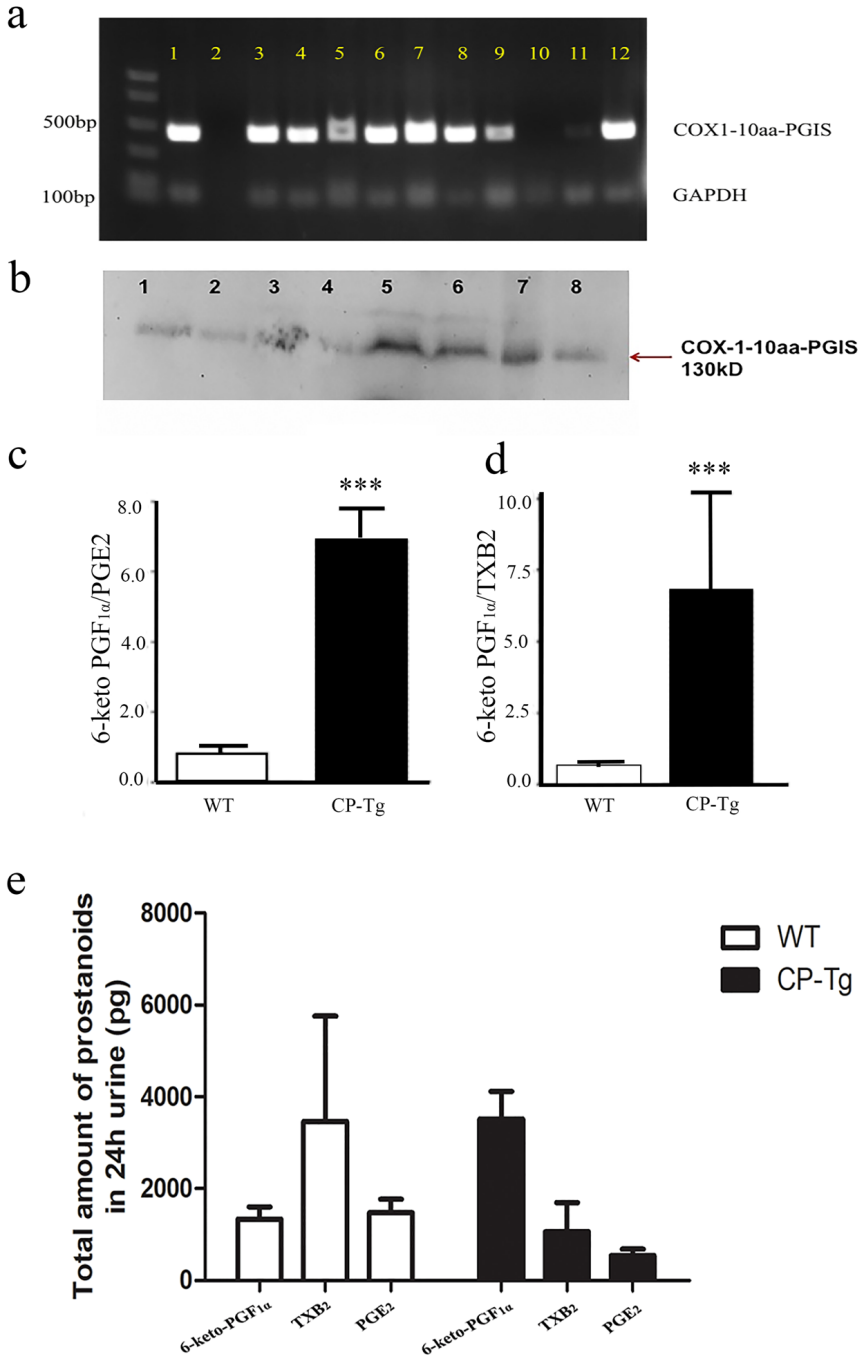
Gene and protein expression in different organs. (**a**) PCR analysis of the organ profile of the COX-1-10aa-PGIS gene in CP-Tg mice. PCR was performed using the different organs of CP-Tg mice. An arrow indicates the 445-bp COX-1-10aa-PGIS. Lanes: 1) positive control, 2) negative control, 3) lung, 4) adipose tissue, 5) intestine, 6) brain, 7) heart, 8) liver, 9) pancreas, 10) wild-type tail, 11) uterus, 12) TG tail. (**b**) Western blot analysis of the different organs of the transgenic mice. The arrow indicates COX-1-10aa-PGIS protein expression at 130 kDa. Lanes: 1) lung, 2) heart, 3) intestine, 4) pancreas, 5) adipose tissue, 6) brain, 7) uterus and 8) liver. The blot was chopped to increase visualization, and the initial blot is attached as Supplementary Fig. 2. (**c**–**e**) LC/MS analysis of AA metabolites, prostanoids in urine of the CT-Tg and wild-type mice. (**c**) Ratio of 6-keto-PGF_1α_ to PGE_2_ in both TG and WT mice. (**d**) Ratio of 6-keto-PGF_1α_ to TXB_2_ in both CP-Tg and WT mice. The above graph represents the mean ± SD (p < 0.001). (**e**) Amount of 6-keto-PGF_1α_, TXB_2_, and PGE_2_ in 24 h urine. The values are represented as mean ± SD.

The expression of individual COX-1 and PGIS were tissue-specific. COX-1 was relatively widely expressed, but PGIS was largely localized in vascular endothelial cells and smooth cells. It was of interest to observe the tissue specificities of the hybrid gene and protein after combining COX-1 and PGIS in one molecule using the pCMV promotor for general expression. Therefore, to evaluate anatomical changes in CP-Tg mice, the major organs were isolated: heart, lung, kidney, brain, and liver. The distribution of the COX-1-10aa-PGIS gene in different tissues of CP-Tg mice was identified by PCR using specific primers as described above. Figure 2a shows the permanent distribution of the COX-1-10aa-PGIS gene according to the different organs. Importantly, the gene was detected in the heart, kidney, and brain, each of which benefitted from the vascular protection provided by the hybrid enzyme. The protein expression of COX-1-10aa-PGIS enzyme in major organs was further identified by Western blot analysis (Fig. 2b). It should be noted that COX-1-10aa-PGIS has a unique molecular mass, 130 kDa, which could be easily distinguished from endogenous COX-1 and PGIS. Similar to the gene distribution profile, COX-1-10aa-PGIS protein was expressed in the major organs: heart, lungs, brain, and kidneys. This result suggested that the single active COX-1-10-aa-PGIS gene could be a potential protector against ischemic heart disease, kidney failure, and stroke caused by thrombosis and vasoconstriction. However, none of the organs showed any gross anatomical difference as observed in CP-Tg mice (Supplementary Fig. 3). Additionally, we also examined the gene and protein expression of COX-1-10aa-PGIS in other organs, except for those major ones. The gene expression was detected in all other organs, and most organs also expressed the protein, except pancreases. Together, these data suggested that our hybrid enzyme COX-1-10aa-PGIS had been successfully integrated into the mice.

### Vascular protection by dual effects through increasing endogenous levels of PGI_2_ and decreasing levels of thrombotic factor TXA_2_ in CP-Tg mice

The catalytic activity of the expressed COX-1-10aa-PGIS in the CP-Tg mice was investigated by the determination of AA metabolite in mouse urine using LC/MS analysis. The ratio of the metabolites of AA to PGI_2_ (final inactive product is 6-keto-PGF_1α_) to PGE_2_ and TXB_2_ (inactive metabolite of TXA_2_) was 7 in the CP-Tg mice and 1 in the wild-type mice (Fig. 2c,d). We also showed the amount of 6-keto-PGF_1α_, TXB_2_, and PGE_2_ in 24 h urine (Fig. 2e) 4. The results revealed that the incorporated COX-1-10aa-PGIS gene into mice exerted multiple effects on the vascular circulation *in vivo*: a) increased PGI_2_ biosynthesis, b) reduced thrombotic TXA_2_ production, and c) decreased inflammatory PGE_2_ biosynthesis. This triple effect could result in a synergetic vascular protection against thrombotic and vasoconstrictive damages from heart diseases *in vivo*.

### Identification of the vascular protective potential through anti-coagulation/anti-thrombosis activity of the single COX-1-10aa-PGIS gene in transgenic mice

**Figure 3:**
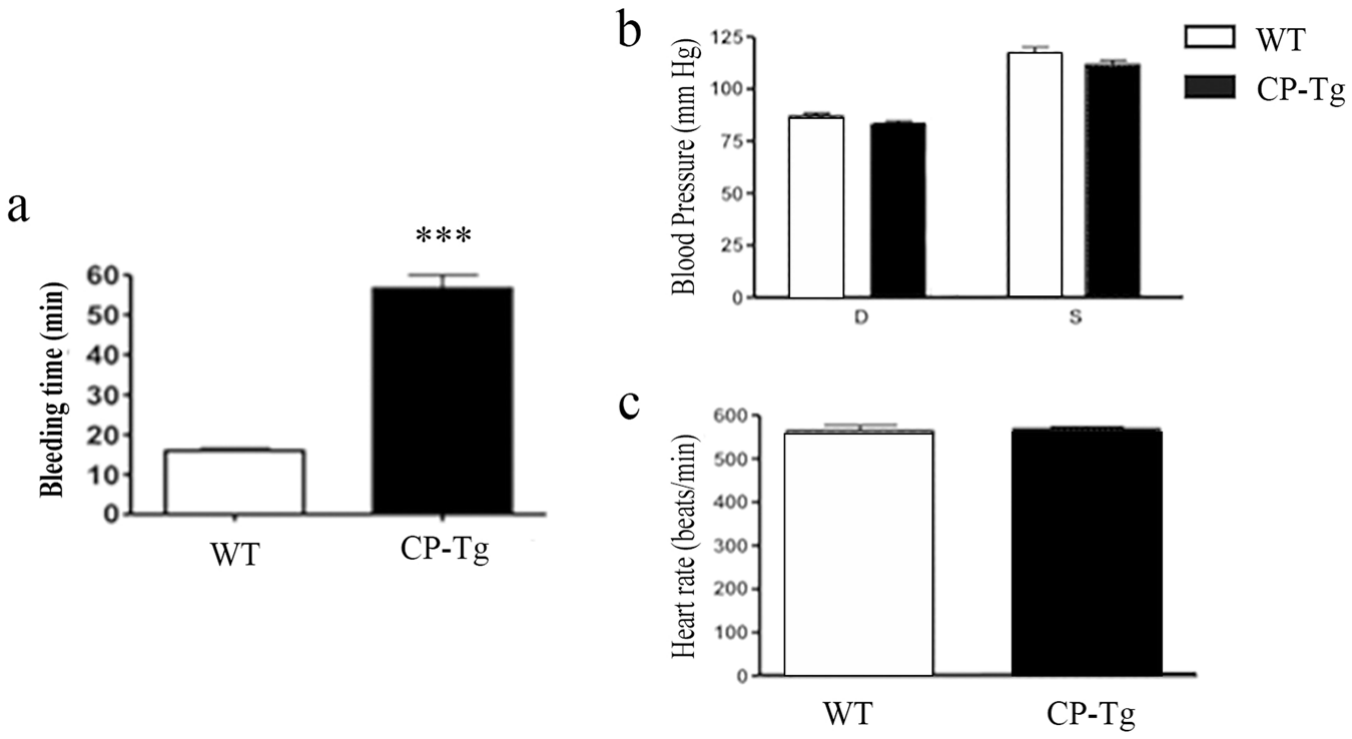
Vascular protective potential assessment. (**a**) Quantification of bleeding time in CP-Tg and WT mice. The bleeding time averaged 55 minutes in CP-Tg and 15 minutes in WT mice (n = 5). (**b**) Blood pressure measurement by the tail cutting method. Both systolic and diastolic blood pressure was measured in TG and WT mice. (**c**) Heart rate measurement by the tail cuff method. Heart rate was measured along with blood pressure in both TG and wild-type mice. The values shown are the mean ± SD (n = 7, p < 0.001). The data were analyzed by the student’s t-test.

One of the major vascular protective characteristics of increased PGI_2_ with decreased TXA_2_ is anti-coagulation/anti-thrombosis. Thus, bleeding time was used as an indication to detect the vascular protective potential of the COX-1-10aa-PGIS gene in the transgenic compared with normal WT mice. Supplementary Fig. 4 shows a representative picture of how the bleeding time was measured using filter paper, which is described in Material and Methods. The bleeding time was 55 mins (n = 6) for CP-Tg mice, which was 3.7-fold longer than that of wild-type mice with an average bleeding time of 15 mins (Fig. 3a). However, no blood cells or oxidized hemoglobin were found in the urine and stool of CP-Tg mice, which indicated bleeding tendencies in the gastric system, intestinal system, and urinary tract were unlikely in the CP-Tg mice. Another possible change caused by the up-regulated PGI_2_ production may be reduced blood pressure. This was not the case for the CP-Tg mice. Their blood pressure, both systolic and diastolic (Fig. 3b), along with heart rate measurement (Fig. 3c) of both TG(+/+) and WT mice, showed no significant differences. In addition, blood counts, cholesterol, and glucose levels were within the normal ranges observed in wild type (data not shown).

### CP-Tg mouse resistance to the induced thrombotic reaction using carotid artery thrombosis as a model

**Figure 4:**
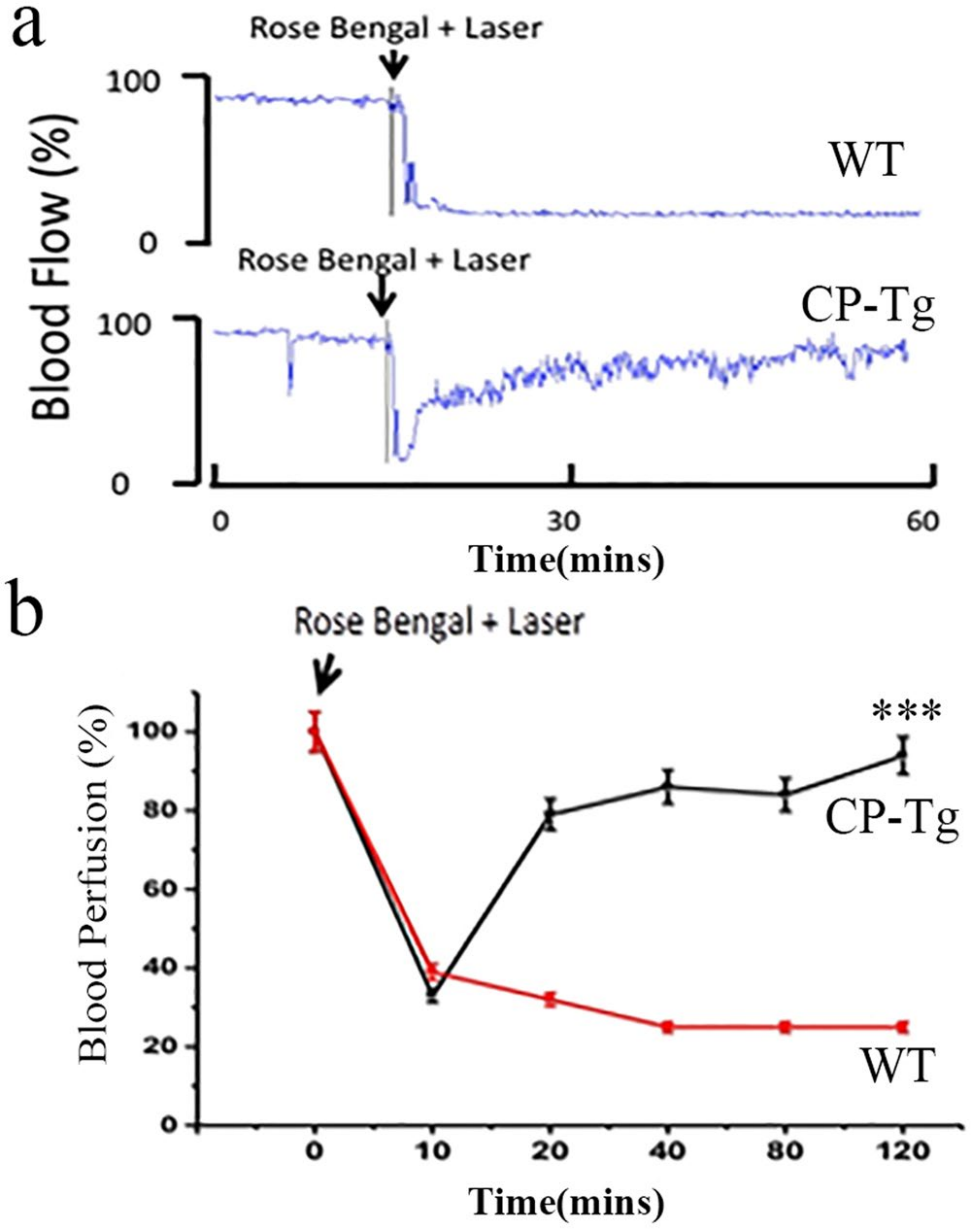
CP-Tg mouse resistance to Rose Bengal/laser beam-induced thrombotic stroke and death. (**a**) Real-time monitoring of carotid artery blood flow before and after Rose Bengal/laser-induced thrombotic occlusion of carotid arteries in a wild-type (top) and a CP-Tg mouse (bottom). The percentage change in reperfusion was calculated at different time intervals and plotted over time. Red squares and black squares indicate changes in perfusion in WT and TG mice, respectively. Rose Bengal (50 mg/kg) was injected through the tail vein, and the time of injection was considered as zero minutes. Blood reperfusion was recorded for a period of 120 minutes. (**b**) Average curves from the two groups of mice obtained using the identical method described in panel A (n = 10, p < 0.01). The data shown are the mean ± SD. The data from the last time point were analyzed by the student’s t-test.

The vascular protective effect on vascular disease caused by permanent COX-1-10aa-PGIS gene expression was demonstrated in a carotid artery thrombosis model. Using this model, both CP-Tg and WT mice were tested for resistance to thrombosis, one of the major leading causes of stroke and heart arrest. The thrombotic obstacle of the carotid artery was induced by photothrombotic ischemia using the combination of Rose Bengal injection, followed by a laser beam light targeting the local artery. Typical real-time monitoring of the blood flow in the occluded carotid arteries of wild-type (Fig. 4a, top panel) and CP-Tg (Fig. 4a, lower panel) mice are shown. The blood flow was not reversibly shut down within 5 minutes, leading to death within a couple hours in wild-type mice (Fig. 4a, top panel). By contrast, blood flow, which was reduced to 90% at the beginning of the induced thrombosis in the artery, quickly recovered to 50% within a few minutes, and then to 70, 80, and 90% within a couple hours in CP-Tg mice (Fig. 4a, bottom panel), which survived the procedure. Further studies with 10 animals in each group provided consistent results (Fig. 4b). The average induced thrombosis in the carotid artery associated reduced blood flow started within minutes and progressed within 2 hours in wild-type mice (Fig. 4b red squares, n = 10). However, in CP-Tg mice, the decreased blood flow during the first few minutes was reversed to 80% normal flow at 20 minutes, and completely recovered after 2 hours, avoiding animal death (Fig. 4b, black squares). These results suggested that CP-Tg mice resisted thrombotic stroke and death induced by Rose Bengal/laser-induced carotid artery thrombosis.

### CP-Tg mouse resistance to death by AA-induced stroke and cardiac arrest

**Figure 5:**
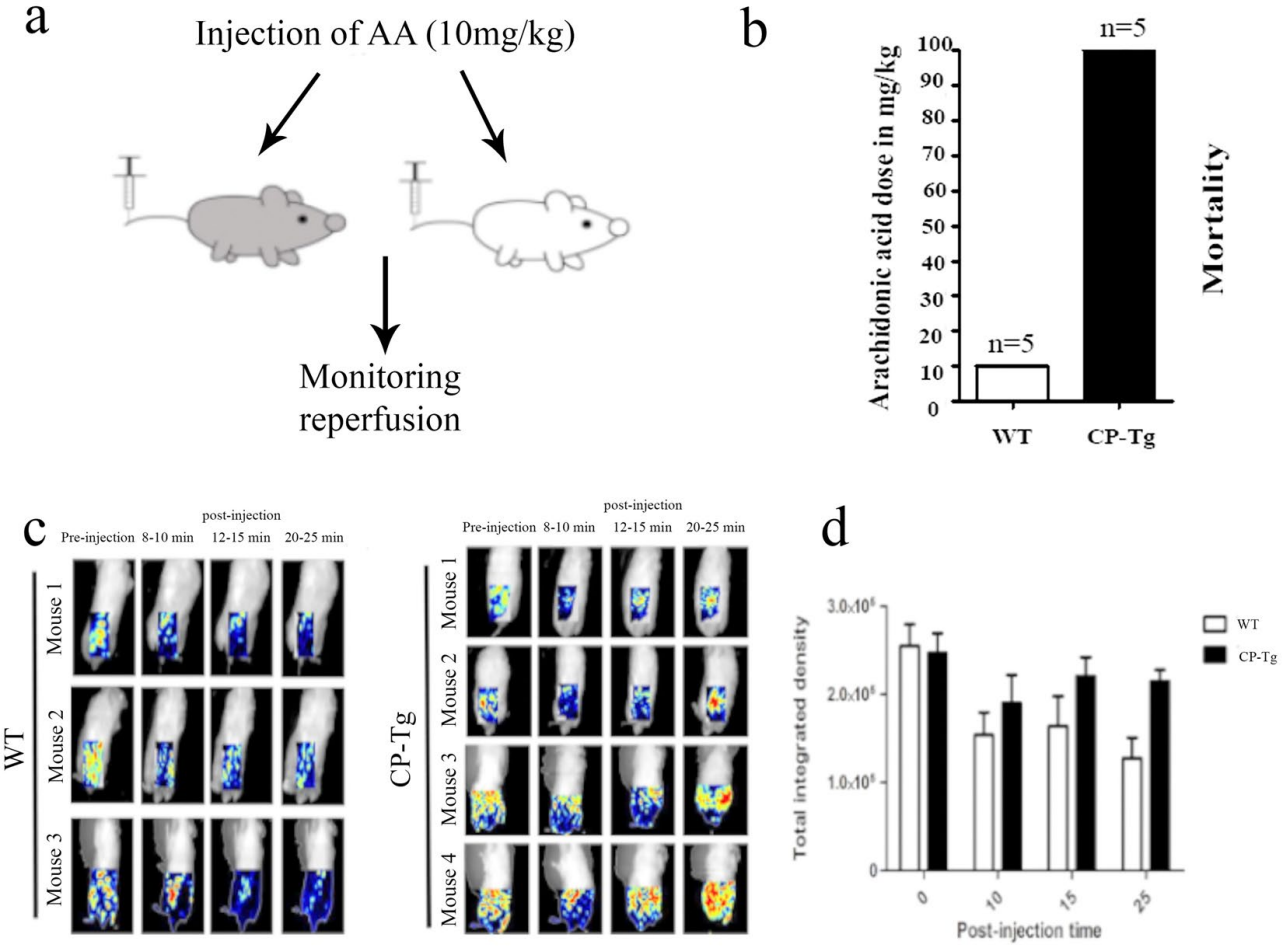
CP-Tg mouse resistance to AA-induced thrombotic stroke, heart arrest and death. (**a**) Diagram of the model of AA-induced thrombotic stroke. (**b**) The mortality of CP-Tg and WT mice following AA injection. The dose of arachidonic acid is the highest dose that each type of mouse could withstand. N = 5 for each type of mouse. (**c**) Peripheral blood reperfusion of CP-Tg and WT mice under AA-induced thrombotic stroke. Blood reperfusion of the lower body of the mice was monitored by image scanning using a laser Doppler before and after injection of AA. Three WT mice and four CP-Tg mice were tested. (**d**) The total integrated density of blood flow analyzed using the ImageJ Heatmap plugin. Data were analyzed by two-way ANOVA with both the time factor and genotype factor. P = 0.015 for the time factor, and p = 0.025 for the genotype factor. There was no significant interaction between the two factors.

An additional thrombotic stroke model was created by overproducing TXA_2_ in the circulation using intravenous (IV) injection of the TXA_2_ precursor AA (Fig. 5a). Due to the enrichment for COX-1 and TXA_2_ synthases in platelets, the infused AA (from IV) could quickly lead to enhanced synthesis of TXA_2_ and trigger platelet aggregation and vasoconstriction, which could a) immediately induce reduced blood circulation in the brain and cause stroke; and/or b) block the coronary artery blood supply and cause cardiac arrest. All wild-type mice suffered from thrombotic stroke and heart arrest after infusion of an average of 10 mg/kg of AA (LD50 = 5 mg/kg, Fig. 5b) within 30 minutes. However, thrombotic stroke and cardiac arrest leading to death was dramatically delayed in CP-Tg mice until they were given an average of 100 mg/kg of AA (LD50 = 50 mg, Fig. 5b). To evaluate the resistance of CP-Tg mice to the induced stroke, a time course of reperfusions of AA-induced ischemia and stroke was monitored using a laser Doppler image system (Fig. 5c). The CP-Tg mice showed initial ischemia for the first 10 minutes, followed by rapidly reperfusion to almost normal blood flow at 25–30 mins during AA (10 mg/kg) induction (Fig. 5c). By contrast, wild-type mice suffered from AA-induced ischemia and were unable to perform the reperfusion, leading to permanent ischemia and death (Fig. 5c). Quantitative data of the integrated intensity are displayed in Fig. 5d. Similarly, the brain anatomy also confirmed that the CP Tg-mice maintained normal blood reperfusion and did not develop vascular damage under 10 mg/kg AA induction at the end of the experiment (2 hours). However, ischemic and vascular damage were clearly observed in wild-type mice under identical conditions (Supplementary Fig. 5). Our results indicated a remarkable protection against stroke and cardiac arrest by the permanently incorporated single hybrid enzyme gene in transgenic mice.

### CP-Tg mice are resistant to non-prostanoid-related vasoconstrictive damage

**Figure 6:**
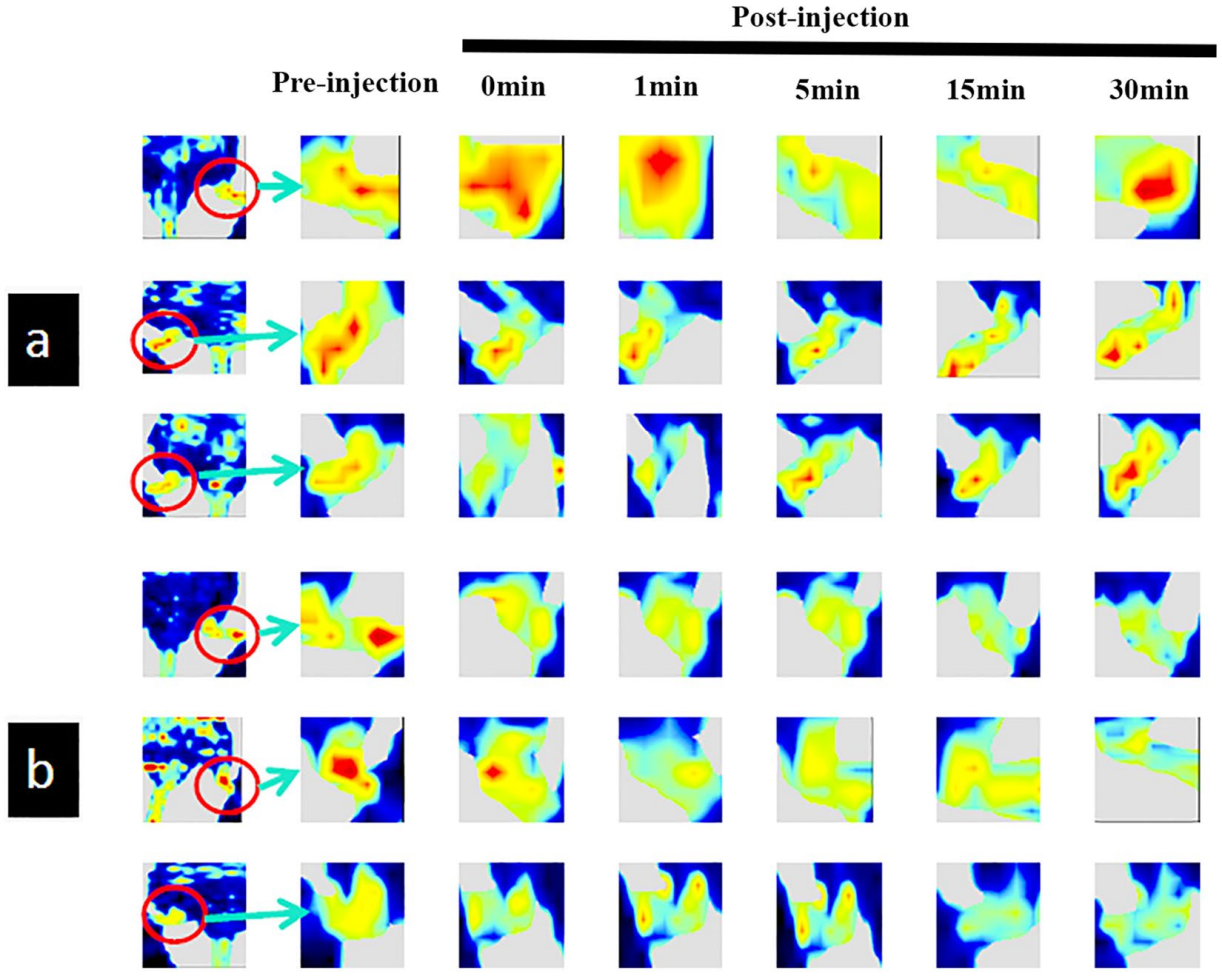
CP-Tg mouse resistance to angiotensin-induced vasoconstrictive ischemia during peripheral blood reperfusion. Blood reperfusion of the hind limbs of the mice was monitored by image scanning using a laser Doppler before and after injection of angiotensin as described in experimental procedures. (**a**) Three CP-Tg mice. (**b**) Three wild-type mice. The green, yellow and red colors represent increasing intensities of blood reperfusion: week, medium and strong, respectively.

To evaluate the resistance of CP-Tg mice to any non-prostanoid-related vascular damage, angiotensin-induced vasoconstrictive ischemia was used. In general, angiotensin can cause vascular stress by inducing acute vascular constriction, which results in reduced blood flow in peripheral and major organs, such as brain, heart, and kidneys. The changes in peripheral blood reperfusion in the hind limbs of mice were monitored by laser Doppler scanning before and after administering angiotensin to the animals. CP-Tg mouse resistance to induced vasoconstrictive ischemia was clearly observed (Fig. 6). All three CP-Tg mice showed slightly decreased reperfusion from 2–15 minutes, followed by recovery to almost normal reperfusion at 30 minutes after receiving the angiotensin (Fig. 6a). In comparison, the reperfusion of all three wild-type mice was dramatically reduced starting at the first minute up to the thirtieth minute without recovery after angiotensin injection (Fig. 6b). These results indicated that the incorporated COX-1-10aa-PGIS gene in transgenic animals was able to resist the induced vascular damage.

### CP-Tg mice are resistant to high-fat diet-induced lipid accumulation

**Figure 7:**
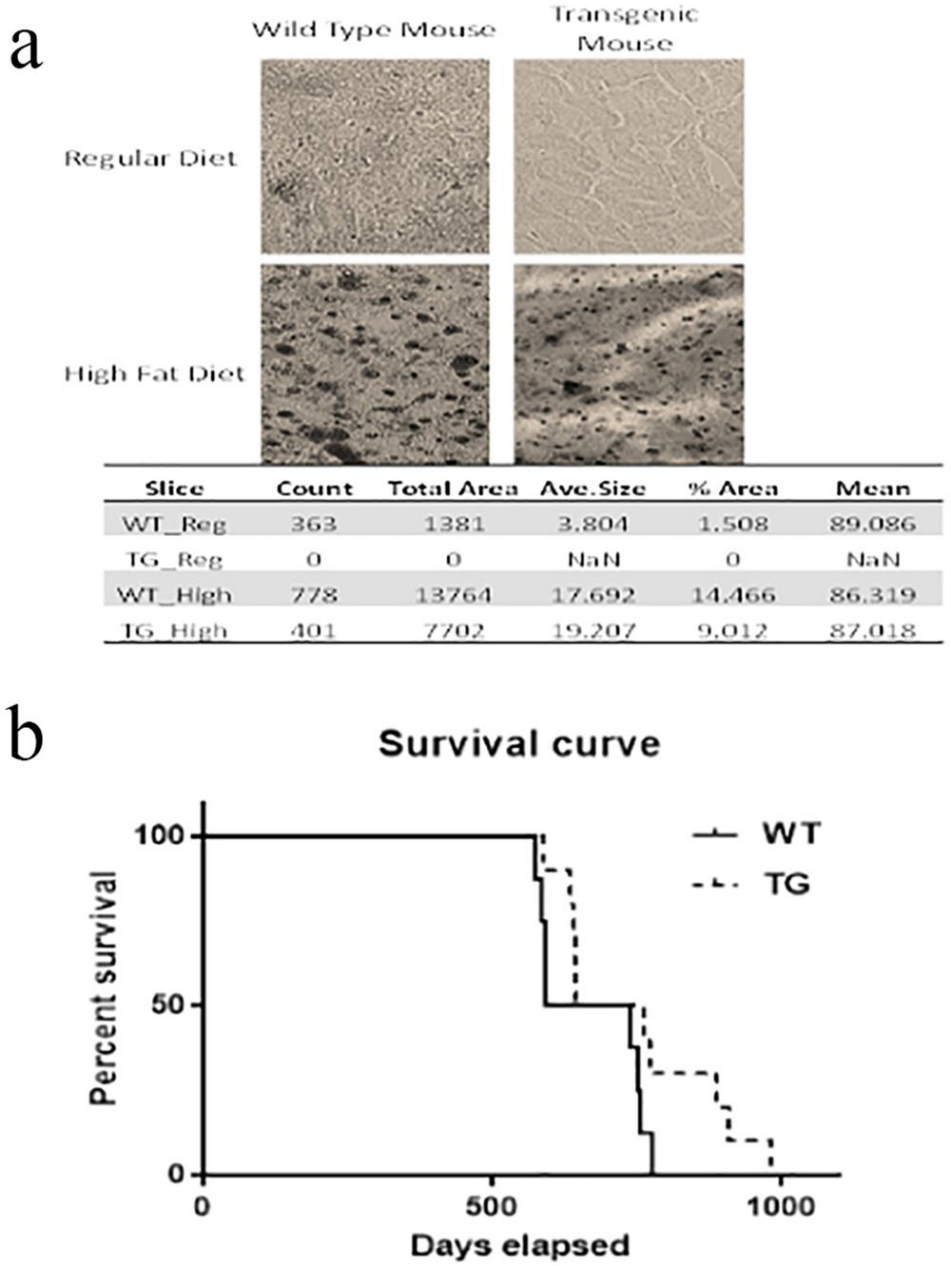
CP-Tg mouse are resistant to a high-fat diet and have a longer lifespan. (**a**) Comparison of lipid deposits between CP-Tg and wild-type mice after being fed a high-fat diet. (**b**) Comparisons of life span between FVB/N-WT and FVB/N-TG mice. Kaplan-Meier survival curves were obtained by recording the life span of the mice from the date of birth to death or termination, as compared with FVB/N-WT mice (solid line, n = 8) and FVB/N-TG mice (dashed line, n = 9). The causes of death included tumor size >1.5 cm (TG 2), emaciation (TG 2), respiratory issues (WT 1, TG 3), or unknown (WT 7, TG 3).

A high-fat diet was fed to the CP-Tg and wild-type mice for 6 months. Anatomic analyses were performed to compare lipid accumulation in liver. Figure 7a shows much less lipid accumulation in the liver of CP-Tg compared with wild-type mice. We used particle analysis in ImageJ to examine the lipid accumulation in liver, in which the dark spots represent lipid accumulation. The count is the number of dark spots, while the total area and the % area indicate the intensity of the dark spots. Additionally, the average size indicates the average particle size, and the mean is the mean intensity of the entire image. Because of the similar average particle size and image background, the lower total area and % area observed in our Tg mice after a high-fat diet suggested a reduction of hepatic lipid accumulation. These data further indicated that the controlled AA metabolites favoring PGIs and disfavoring PGE_2_ and TXA_2_ have beneficial resistance effects against high-fat diet-induced damage to the liver.

### CP-Tg mice have a long lifespan

The FVB/N CP-Tg mice and wild-type FVB/N-WT mice were maintained in identical life conditions, such as a stable temperature at approximately 24 °C with identical diets and general health care. Their overall lifespan was recorded for almost three years. The CP-Tg mice were not only free from suffering bleeding tendencies, headaches (no head-directed wiping and scratching^17^), hypotension, and other vascular-related dysfunctions correlated with the progression of aging, but they also had longer lifespans in general (Fig. 7b), in which the longest life span reached almost 1000 days, with an average of 757.6 ± 40.63 days. Wild-type control mice had a longest lifespan of 670.5 ± 32.37 days (Fig. 7b).

## Discussion

For decades, the use of PGI_2_ mimic to treat vascular diseases, such as pulmonary arterial hypertension, has been well documented^18,19^. The vascular protective effects of PGI_2_ have also been well demonstrated by studies showing an increase in the risk of heart attack associated with the use of COX-2 inhibitors, such as Vioxx, by suppressing PGI_2_ production. By contrast, reducing TXA_2_ production, such as through aspirin use, is a more traditional way to provide protection heart disease and stroke. Anti-inflammatory and anti-proliferation drugs are often used to treat vascular inflammation during restenosis and arteriosclerosis development through suppression of the biosynthesis of an inflammatory factor, such as PGE_2_. Thus, it becomes very interesting to develop an ideal intervention that could integrate the three forms, increasing PGI_2_ biosynthesis associated with a decrease in TXA_2_ and inflammatory PGE_2_ production *in vivo* to enhance the protective power against heart disease and stroke. Our CP-Tg mouse is the first transgenic mouse line used to test this intervention. As expected, the single COX-1-10aa-PGIs gene incorporated into the mice redirected the endogenous AA metabolite in favor of PGI_2_ production while reducing TXA_2_ and PGE_2_ in the circulation, as evidenced by LC/MS analysis (Fig. 3). In particular, functional COX-1-10aa-PGIS enhanced expression in major organs, such as the heart, brain, and kidneys, suggesting that the blood circulation and oxygen level might also be increased in these organs. This phenomenon could provide a fundamental benefit by preventing vascular diseases caused by acute thrombotic and vasoconstrictive damages, as well as chronic ischemia.

By contrast, there were no indications of any abnormal bleeding or hypotension, which might have resulted from the over-expression of COX-1-10aa-PGIS in transgenic mice. Although bleeding time was prolonged in CP-Tg mice due to the anti-thrombotic properties, it did not cause any bleeding disorders and remained within a safe range. PGI_2_ is a potent endogenous vasodilator and may therefore lead to blood pressure lowering similar to the administration of a PGI_2_ mimic. However, both the systolic and diastolic blood pressures of the CP-Tg mice were similar to those of wild-type mice using the tail cuff method (Fig. 5). This phenotype could be explained by compensatory mechanisms that regulate blood pressure to normal levels in CP-Tg mice, which is a common physiological effect when using the transgenic approach instead of drug therapy.

Thrombosis and vasoconstriction are major causes of death by stroke and cardiac arrest. The CP-Tg mice showed resistance to ischemia and death in the Rose Bengal/laser- and AA-induced thrombotic stroke and cardiac arrest models (Figs 4, 5), as well as resistance to angiotensin-induced vascular damage (Fig. 6). These data provide strong evidence that the single COX-1-10aa-PGIS gene has preventive and therapeutic value against thrombotic/vasoconstrictive heart disease and stroke in general.

Furthermore, it should be noted that a mental balancing test of the CP-Tg mice revealed better learning memory and less anxiety^16^ compared with the control mice. In addition, all the heterozygous and homozygous mice shared equal phenotypes and displayed normal behavioral patterns. Altogether, these findings provide insight suggesting that up-regulated PGI_2_ and down-regulated TXA_2_ and PGE_2_ via the transgenic approach using the COX-1-10aa-PGIS gene have notable major adverse effects *in vivo*. The longer lifespan of the CP Tg mice (Fig. 7) provides initial data to justify our conclusion. The observed resistance to high-fat diet–induced lipid accumulation in CP-Tg mouse liver further demonstrated the possible long-term benefits of the prevention of heart diseases by controlling the microenvironment of cellular AA metabolism in favor of PGI_2_ using the COX-1-10aa-PGIS gene.

An ideal transgenic approach for expression of a therapeutic gene *in vivo* is to maintain the ability to deactivate the gene after the desired benefit has been achieved. In our future studies, we would like to test if COX-1-10aa-PGIS activity in transgenic mice can be controlled by inhibiting PGI_2_ production activity, especially by the use of COX-1 and PGIS inhibitors, such as aspirin and U-51605^20^. The availability of such options allows control of the incorporated COX-1-10aa-PGIS activity in the transgenic mice. This will be examined in our future studies.

In conclusion, the COX-1-10aa-PGIS gene composed of a single enzyme with unique triple catalytic activities has been successfully incorporated into transgenic mice, which could control the microenvironment of cellular AA metabolites to provide triple vascular protection activities by increasing the conversion of AA to PGI_2_ while reducing the levels of AA available for TXA_2_ and PGE_2_ synthesis. The resistance to the induced thrombotic and vasoconstrictive damage caused by the incorporated COX-1-10aa-PGIS was clearly identified *in vivo*. Our approach represents a new vascular protective strategy because triple effects have not been successfully achieved by any drugs to date, such as aspirin or PGI_2_ mimics. Thus, our findings raise the possibility that heart disease may also be treated in the future by a simple transgenic approach targeting specific organs, when ethical and safety issues have been overcome and perfected accordingly.

## Materials and Methods

### Animals

The CP-Tg, containing a hybrid enzyme of COX-1 linked to PGIS (COX-1-10aa-PGIS), and WT mice used in the experiments were maintained in the Animal Care Facility at the University of Houston. The animals were kept in groups of one to five mice per cage in a room with a 12-h light cycle (lights on at 6:00 AM and off at 6:00 PM) at 23 °C with food and water available ad libitum. Male and female mice were randomized across the groups, with each group having an even number of male and female mice. All experiments were approved by and conducted in accordance with the University of Houston and the University of Texas M.D. Anderson Cancer Center Institutional Animal Care and Use Committees (IACUC) and implemented following the National Research Council’s Guide of The Care and Use of Laboratory Animals.

### Engineering of the SCEC, COX-1-10aa-PGIS cDNA and subcloning

The sequence of COX-1 linked to PGIS through a 10-amino acid residue (10 aa) linker (COX-1-10aa-PGIS) was generated by PCR and subcloning procedures provided by the vector company (Invitrogen) as previously described^11,12,17,21^. The resulting cDNA was successfully subcloned into the pcDNA 3.1 vectors at Kpnl and BamHl sites containing a cytomegalovirus early promoter by PCR. The correct cDNA sequences were confirmed by DNA sequencing and restriction enzyme digestion analyses.

### Construction of the cDNA plasmid to generate transgenic mice over-expressing COX-1-10aa-PGIS

The pcDNA3.1 vector containing COX-1-10aa-PGIS was cut at Nrul and Sphl sites to obtain a 3.2-kb cDNA of COX-1-10aa-PGIS with a pCMV promoter and PGH polyadenylation site (pA) as a transgene fragment. The identity of the construct was further confirmed by restriction enzyme mapping and DNA sequence analysis.

### Generation of transgenic mice overexpressing COX-1-10aa-PGIS

FVB/N (Jackson Lab) female mice were maintained under a 14-hr light/10-hr dark cycle and superovulated with 5 IU of pregnant mare serum-gonadotropin followed by (after 48 hrs) 5 IU of human-chorionic gonadotropin. These females were placed with intact FVB males for mating. Fertilized oocytes were collected from the isolated oviducts of the superovulated females the following morning and treated briefly with bovine hyaluronidase in M2 medium to remove the associated debris. The COX-1-10aa-PGIS gene fragment was gel-purified using a QIAGEN kit, diluted (immediately before microinjection) to a concentration of 3 ng/μL and then microinjected into the pronuclei of fertilized oocytes using a pronuclear microinjection technique. The manipulated oocytes were transferred surgically into the oviducts of surrogate (pseudo-pregnant) recipient female mice and allowed to develop to full term to obtain the new generation of mice, which was tested for the presence of the COX-1-10aa-PGIS gene (CP-Tg). The initial procedures were performed at the University of Texas M. D. Anderson Cancer Center (Houston, TX).

### Identification of the COX-1-10aa-PGIS gene in mice

To evaluate the expression of COX-1-10aa-PGIS in the new mice, PCR analysis was performed using mouse tail tissue. Briefly, tails (1.5–2 mm) were digested in 100 μl of 1 × PCR buffer with detergents (0.45% NP40, 0.45% TWEEN 20) and 10 μl proteinase K (10 mg/ml) at 60 °C overnight. The sample was further denatured in proteinase K by boiling for 15 min, followed by cooling on ice for 5 minutes. The isolated genomic DNA (2 µL tail DNA was added to a final reaction volume of 20 µL) was subjected to PCR analysis using PCR Reaction Buffer (1× PCR buffer, 2.5 mM MgCl_2_, 200 μM dNTPs, 1 μM each designed primer, and 1 unit Taq Polymerase under the following conditions: hold at 94 °C for 4.5 min, 30 cycles of 94 °C for 30 sec, 72 °C for 1 min, and holding at 4 °C until analysis. The primer sets used for the COX-1-10aa-PGIS gene were as follows: sense 5′-CCTCAAGGGTCTCCTAGGGA-3′ and antisense 5′-GTGCTTCTCCTTCATCCTCGT-3′. The amplified product with a length 445 bp encoded the unique region (from the COX-1 C-terminal region and the 10-aa linker to the PGIS N-terminal region) of the COX-1-10aa-PGIS hybrid enzyme. The PCR product was analyzed by agarose gel electrophoresis.

### Determination of the protein expression of COX-1-10aa-PGIS in mouse tissues

The tissues were homogenized in PBS on ice and then immediately solubilized in SDS-PAGE buffer containing 2% SDS and analyzed by 7% SDS-PAGE and Western blotting as described previously^11–13,17^.

### Determination of the endogenous AA metabolite profiles in mice using urine samples by LC/MS

The mouse urine was collected (n = 6) and passed through a C18 cartridge. The eluent (eluted with acetone) was dried under nitrogen gas, and the residual substrate was diluted in buffer A (containing 0.1% acetic acid and 35% acetonitrile). The prepared sample was injected into the Waters Micromass LC/MS/MS system using an auto-sampler, in which the metabolites were first separated using a RP-HPLC C18 column and then automatically injected into the mass detector equipped with an ESI source in negative mode. Synthetic AA, PGE_2_, 6-keto-PGF_1α_ (PGI_2_ stable and final metabolite), and TXB_2_ obtained from Cayman Chemical Company (Ann Arbor, MI), were used as standards to calibrate the LC/MS system, identify the corresponding prostanoids, and normalize the detection limits and sensitivities.

### Bleeding time assay

The tip of the mouse tail was clipped (2 mm) with scissors, and blood was blotted onto filter paper while the time was noted at the first seconds. Thereafter, the clipped tip of the tail was blotted on a clean section of filter paper every 30 seconds until there was no longer detectable blood, at which point the total bleeding time was determined according to the following formula:

$${\rm{Bleeding}}\,{\rm{time}}\,({\rm{\min }})={\rm{number}}\,{\rm{of}}\,{\rm{dots}}\times 0.5$$

The bleeding time was compared between wild-type and transgenic mice.

### Blood pressure and heart rate detection

The blood pressure (BP) and heart rate (HR) were measured using the tail cuff method using the CODA system from Kent Scientific Corporation, Torrington, CT. Blood pressure was measured for every individual mouse for three consecutive days to allow for acclimatization and thereby obtain more accurate measurements. Systolic, Diastolic BP, and HR were measured and plotted to compare any changes in these parameters between WT and CP-Tg mice.

### Rose Bengal/Laser-induced thrombotic stroke model

The thrombotic stroke model was created by establishing photochemical vascular injury in the carotid artery of CP-Tg mice according to previously described methods [4]. Briefly, male mice were anesthetized with sodium pentobarbital (80 mg/kg, i.p.) and placed in a supine position. Then, the left common carotid artery was exposed, and a laser Doppler flow probe 407 connected to a laser Doppler Perfusion Monitor (LDPM) Unit-Periflux System (PF) 5010 was positioned on the artery to record a constant baseline blood flow. Next, the carotid artery was exposed to 1.5 mW of a green laser beam at 540 nm (Melles Griot, CA, USA) from a distance of 5 cm and Rose Bengal (50 mg/kg) was injected through the tail vein. At this point, blood flow was constantly recorded using the accompanying software. The formation of an occlusive thrombus was indicated by the cessation of blood flow.

### Determination of resistance to AA-induced thrombotic heart arrest *in vivo*

Sodium AA (1 mg/ml, Sigma) in saline solution was continually infused into the mice through the tail vein. Heart beat was monitored using an ultrasound monitor (Sono Site - TITAN). The time at which the mouse heart beat stopped was used as the end-point for the AA resistance test.

### Determination of angiotensin II-induced vasoconstrictive peripheral reperfusion damage

The mice were anesthetized by isoflurane inoculation. Angiotensin II (160 μg) was administered through the tail vein and peripheral blood reperfusion before and after angiotensin injections were monitored by a laser Doppler scanner (Perimed).

### Determination of hepatic lipid accumulation-induced by a high-fat diet

Wild-type and transgenic mice were both fed a regular diet and a high-fat diet (Teklad, TD.01383). The diet was nutritionally complete for mice, composed of the parent 2018 diet with 2% cholesterol. The mice were fed for 6 months. After that, the mice were sacrificed, and their liver were sectioned for hematoxylin and eosin staining.

### Statistical analysis and graphing

Data were analyzed using GraphPad Prism (GraphPad software, Inc., San Diego, USA) with the appropriate analytic methods. Adobe Photoshop CC was used to increase the resolution of the images. All images were created in our laboratory.

## Electronic supplementary material

Supplementary Dataset
