# Supplementary material for: Creating a mouse model resistant to induced ischemic stroke and cardiovascular damage
Source: Sci Rep. 2018 Jan 26;8:1653. doi: 10.1038/s41598-018-19661-y (PMC5786049; doi:10.1038/s41598-018-19661-y)
Supplement: Supplementary file 1 — Supplementary Dataset [file 41598_2018_19661_MOESM1_ESM.docx]

**Supplemental information**

**Creating a mouse model resistant to induced ischemic stroke and cardiovascular damage**

**Qing-Lan Ling**, Anita J. Mohite**, Emma Murdoch**, Hironari Akasaka**, Qun-Ying Li**, Shui-Ping So ** and Ke-He Ruan*,**

The Center for Experimental Therapeutics and Pharmacoinformatics, Department of Pharmacological and Pharmaceutical Sciences, College of Pharmacy, University of Houston, Houston, Texas 77204, U.S.A.

*To whom correspondence should be addressed: Ke-He Ruan, Ph.D., M.D., The Center for Experimental Therapeutics and PharmacoInformatics, Department of Pharmacological and Pharmaceutical Sciences, University of Houston, Room 521 Science and Research 2 Building, Houston, TX 77204-5037, United States. Tel.: + 1 713 743 1771; fax: + 1 713 743 1884; Email: [kruan@central.uh.edu.](mailto:kruan@central.uh.edu)

**Contribution: They contributed equally.


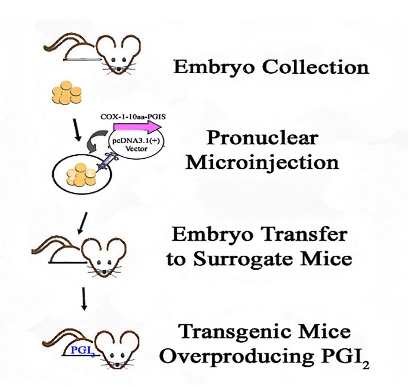


Supplementary Figure 1. Schematic presentation of the steps for the CP-Tg mouse generation used the above cDNA of human COX-1-10aa-PGIS.


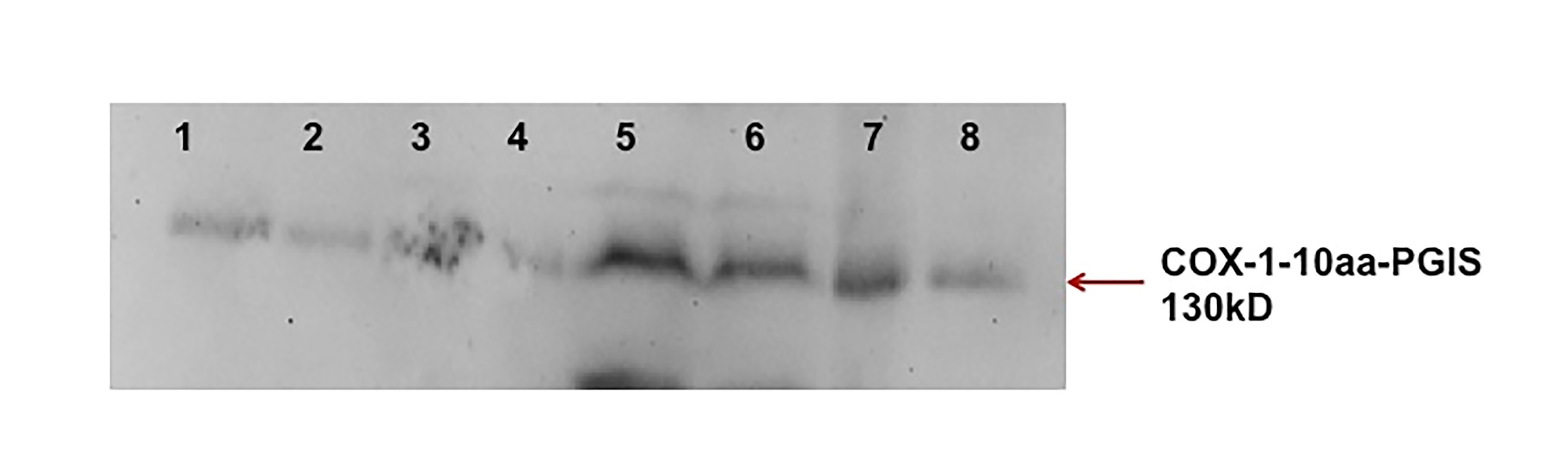


Supplementary Figure 2. Raw western blot pcture for Figure 2b.


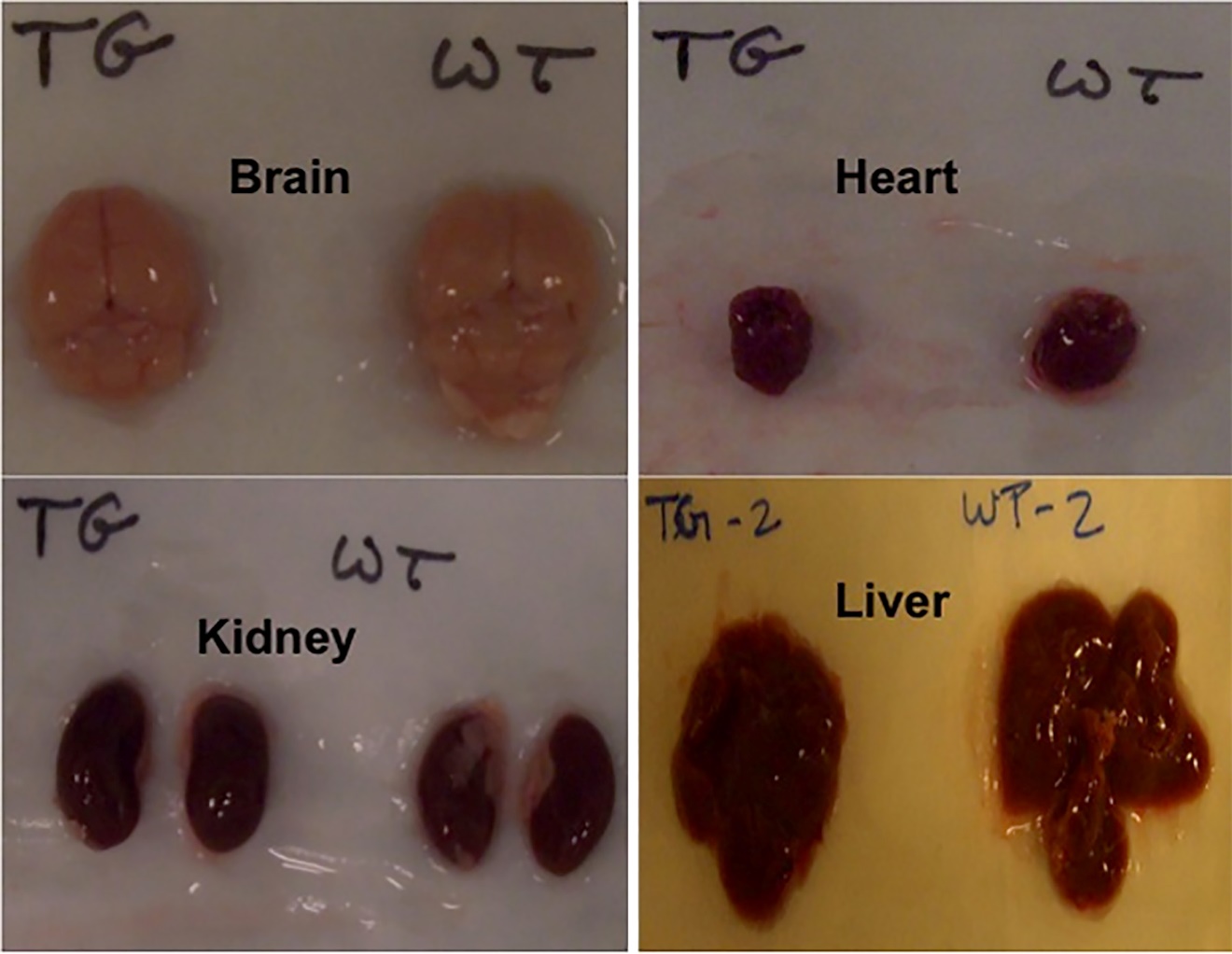


Supplementary Figure 3. Organ pictures of wild type (WT) mice and transgenic (TG) mice


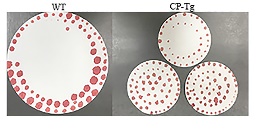


Supplementary Figure 4. Bleeding time measurement in CP-Tg and WT mice. After clipping the tail, blood was blot on filter paper every 30s until bleeding stopped.


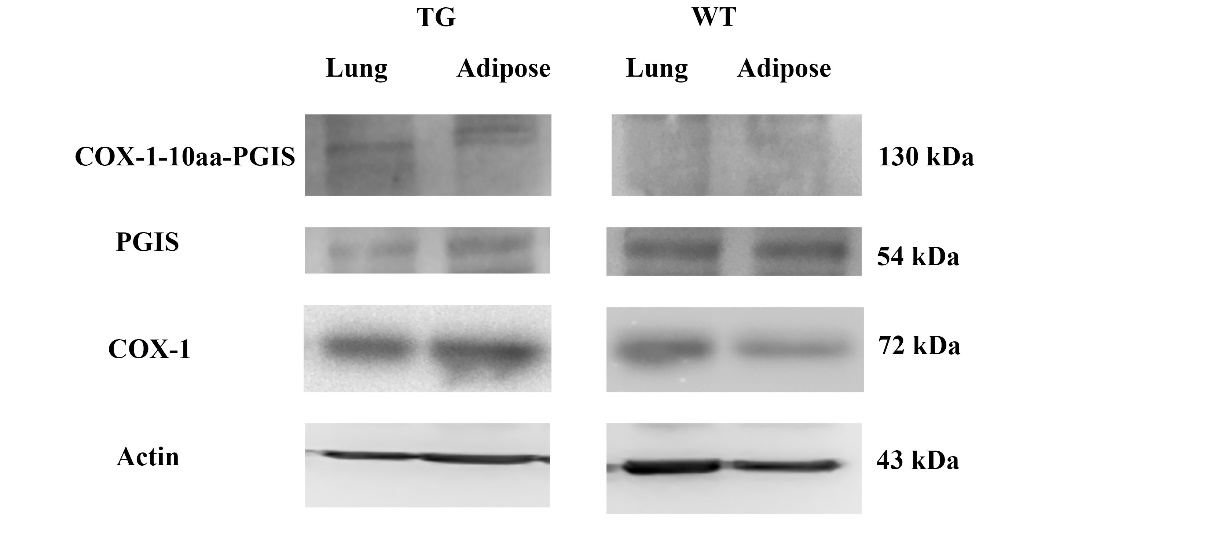


Supplementary Figure 6. Western blots results showing the expression of COX-1- 10aa-PGIS, PGIS, COX-1, and actin from lung and adipose tissue of both wild-type and transgenic mice.


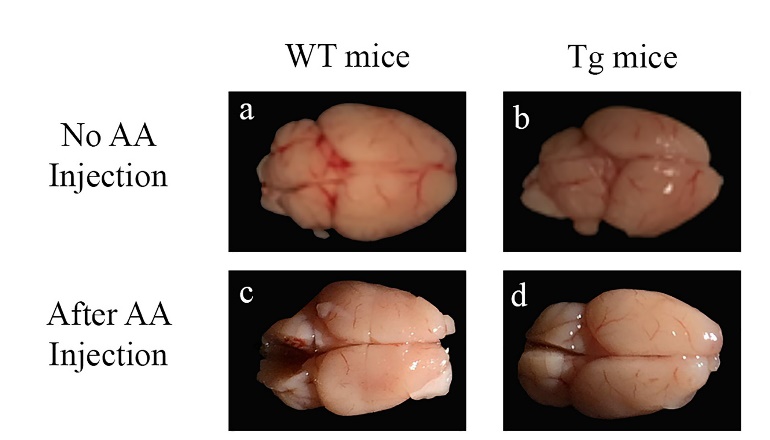


Supplementary Figure 5. Brain pictures in AA-induced thrombotic stroke model.
